# Timescale of a magmatic-hydrothermal system revealed by ^40^Ar–^39^Ar geochronology: the Mio-Pliocene Campiglia Marittima system (Tuscany, Italy)

**DOI:** 10.1038/s41598-022-10867-9

**Published:** 2022-05-03

**Authors:** Gianfranco Di Vincenzo, Simone Vezzoni, Andrea Dini, Sergio Rocchi

**Affiliations:** 1 Istituto di Geoscienze e Georisorse-CNR, via Moruzzi 1, 56124 Pisa, Italy; 2 Dipartimento Scienze della Terra, Università di Pisa, Via S. Maria 53, 56126 Pisa, Italy

**Keywords:** Geochemistry, Petrology, Volcanology

## Abstract

Petrology and timing of magmatic-hydrothermal systems and the linkage between plutonic and volcanic domains are central topics in geosciences, because of broad implications for natural hazards and exploitation of natural resources. We investigated by the ^40^Ar–^39^Ar method the timescale of a well-characterized natural example, the Mio-Pliocene Campiglia Marittima magmatic-hydrothermal system (Tuscany, Italy). ^40^Ar–^39^Ar data from pristine and homogeneous trioctahedral micas and sanidine from the plutonic-hydrothermal-subvolcanic-volcanic sequence (from the Botro ai Marmi Granite to the San Vincenzo Rhyolite) record crystallization ages and define a temporal sequence lasting 973 ± 43 ka, starting from 5.409 ± 0.043 Ma. K-feldspar from mafic and felsic porphyries, unlike micas, are affected by submillimetre, micropore laden, alteration domains consisting of secondary K-feldspar and albite, and yielded staircase-shaped age spectra, compatible with a ternary mixing. Results document that the San Vincenzo Rhyolite consists of two diachronous batches, the first emplaced at 5.0024 ± 0.0062 Ma, closely following emplacement of mafic porphyries, the second at 4.4359 ± 0.0045 Ma. Bulk of hydrothermal deposits, consisting of skarns and associated Zn–Pb(-Ag) mineralization predating Fe–Cu ore, formed within the first ~ 400-ka lifetime of the whole sequence and was closely followed by the first eruption which should have run out most of the ore-forming potential of the system.

## Introduction

Understanding the petrology and timescale of magmatic-hydrothermal systems and the link between plutonic and volcanic domains are key topics in Earth sciences, due to the implications for natural hazards (volcanic eruptions, gas emissions, earthquakes) and for exploitation of natural resources (ore deposits and geothermal resources). Knowledge of accurate and precise timescale and lifetime of an igneous-hydrothermal system and associated ore deposits is crucial in order to develop genetic models. Timescales have been addressed through either thermal modelling^1–4^ or geochronological studies based on radioisotopic techniques (mainly U–Pb data on zircon) on fossil systems^5–8^. From recent literature data on natural examples, it is becoming increasingly clear that igneous-hydrothermal systems are formed by single or episodic events with a relatively short duration, ranging from tens to hundreds of ka^6,9–11^, whose resolution requires high-precision geochronological data.

The ^40^Ar–^39^Ar method (variant of the K–Ar dating technique) represents an invaluable tool to constrain timing and rate of geological processes. Improved precision thanks to a new generation of multi-collector noble gas mass spectrometers^12–15^ together with efforts in increasing accuracy through calibration of mineral reference materials by astronomical tuning^16,17^, has recently allowed the ^40^Ar–^39^Ar method to approach the temporal resolution of the zircon U–Pb CA-ID-TIMS (chemical abrasion-isotope dilution-thermal ionization mass spectrometry), thus generating a renewed and growing interest in the ^40^Ar–^39^Ar dating technique. ^40^Ar–^39^Ar dates are often interpreted to record the cooling below a certain temperature, the “closure temperature”^18^, which in principle would allow a straightforward interpretation of Ar data. This approach is commonly used regardless of microstructural and microchemical arguments, and it is based on the critical assumptions that temperature is the only parameter controlling the rate of argon isotope transport. However, there is growing evidence of the importance in nature of dissolution-reprecipitation processes, which realize at much faster rates than pure volume diffusion, whenever fluids out of equilibrium with a given mineral are present^19–22^, and produce a spatially and temporally inhomogeneous crystal structure. In line with this view, several field-based studies have shown that variation in Ar isotope records can be closely linked to microstructural and microchemical variations^23–27^, thus highlighting the primary role in the transport of Ar isotopes of mineral reactivity and in turn of fluid circulation, and the importance of integrating ^40^Ar–^39^Ar data with mineral-textural and chemical analysis at the microscale.

In this study, we investigate by the ^40^Ar–^39^Ar laser method an exceptionally well-characterized natural example, the Mio-Pliocene Campiglia Marittima magmatic-hydrothermal system of Tuscany^28,29^, and we explore the link of Ar isotope record with microstructural and microchemical variations in datable potassic minerals of plutonic, hydrothermal, sub-volcanic and volcanic rock samples. Results allow to reconstruct in detail the temporal evolution of the magmatic-hydrothermal system investigated, with important implications for the petrogenesis of the associated ores.

## Geological background

**Figure 1:**
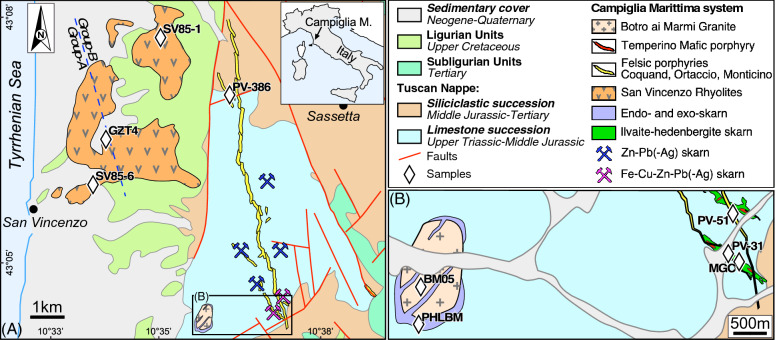
(**A**) Geological map of the Campiglia Marittima area showing the location of the investigated samples modified after Vezzoni et al.^28^. (**B**) Close up of the southern zone of the investigated area. The dashed line separating Group-A from Group-B rhyolites (San Vincenzo Rhyolite) is from Pinarelli et al.^46^. (Figure generated by Canvas x draw v. 7.0.2).

Campiglia Marittima is located in the internal side of the northern Apennine fold-and-thrust belt (Fig. 1), which formed through the collision between the Adria (Africa) and the Sardinia-Corsica (Europe) plates^30,31^. The current architecture of Tuscany resulted from the late Oligocene–middle Miocene compression, followed by the Miocene to present extensional phase^31,32^. The compressional phase produced the stacking of different tectonic units, which in the Campiglia Marittima area includes from the bottom to the top: (1) Tuscan Nappe, (2) Sub-Ligurian units and (3) Ligurian units. The stacked units were then affected by the extensional phase, which produced a thinned continental crust (~ 20–25 km—Piana Agostinetti and Amato^33^), followed by the emplacement of crustal- and mantle-derived magmas (the Tuscan Magmatic Province^34^), ore deposits^35,36^ and diffuse, still locally active, hydrothermal activity (e.g., Larderello-Travale and Mt. Amiata^36,37^). Campiglia Marittima was one of the main centres of the Tuscan Magmatic Province and was affected by multiple magmatic events, which were accompanied by skarn formation and sulphide deposition. A relative chronological sequence was recently reconstructed using field data by Vezzoni et al.^28,29^ and will be used as a reference frame in the present study. Available geochronological data for the Campiglia Marittima magmatic-hydrothermal sequence are limited to a few K–Ar dates^38^, ranging from 5.7 ± 0.16 Ma (K-feldspar of the Botro ai Marmi granite) to 4.3 ± 0.13 Ma (whole rock of a porphyritic dyke), and to single-grain ^40^Ar–^39^Ar total fusion laser analyses of euhedral and clear sanidine crystals^39^ from a Group-B sample of the San Vincenzo Rhyolite (see below), which yielded an emplacement age of 4.41 ± 0.04 Ma (± 2σ, recalculated using an age of 523.98 Ma^40^ for the MMhb-1 reference material to improve consistency with the astronomically-calibrated age of 28.201 Ma of the Fish Canyon sanidine^16^). More recently, a zircon U–Pb age of 5.442 ± 0.012 Ma (± 2σ), based on CA-ID-TIMS data^41,42^, has been reported for the Botro ai Marmi Granite. Available geochronological data therefore constrain the onset of the igneous event in the area at the late Messinian and limit the duration of the whole plutonic-volcanic system to ~ 1 Ma.

### The Campiglia Marittima magmatic-hydrothermal system

The sequence started with the Botro ai Marmi Granite, which intruded a thick sequence of non-metamorphic carbonatic rocks at a depth of a few kilometres (~ 0.10–0.15 GPa) and produced an elongated contact aureole, with temperatures of ~ 500–550 °C^43^ at the top. Two main types of metasomatic bodies followed the contact metamorphism^28^: (1) endo- and exo-skarn, close to the pluton roof; (2) a distal ilvaite-hedenbergite skarn. The endoskarn veins cut the pluton and are interconnected with the overlying exoskarn. The exoskarn occurs as massive bodies replacing the marble at the contact with the pluton^29,42^. The distal skarn forms the main metasomatic bodies and consists of sub-vertical sigmoidal bodies in the eastern side of the thermal aureole, ~ 0.5–1 km above the buried pluton^28^ (Fig. 1) and are associated with Zn–Pb(-Ag) sulphide ores. Several magmatic events followed the formation of the skarns. A first, mafic magma (Temperino porphyry) intruded the distal skarn bodies, forming dikelets and filling residual voids in the skarn. The emplacement of mafic magma induced the overprinting of previous Zn–Pb(-Ag) sulphides by Fe–Cu sulphide ore. Subsequently, several generations of felsic dykes emplaced mainly within the basal carbonatic formations and, to a lesser extent, in the overlying siliciclastic succession (Fig. 1). Three distinct sub-units have been identified^28^: (1) Coquand, (2) Ortaccio and (3) Monticino porphyries. The Coquand porphyry consists of two dikes, cropping out discontinuously for ~ 2 km in length and spatially associated with the distal skarn bodies. The Coquand samples are typically white to grey, to pale green in colour, and include decimetre-sized mafic enclaves. The Ortaccio porphyry consists of a single dyke, cropping out almost continuously for ~ 8 km in length (maximum thickness ~ 20 m) and crosscutting the Coquand dykes. It is characterized by a yellowish colour, abundant centimetre K-feldspar phenocrysts and rare centimetre mafic enclaves. The Monticino porphyry crops out in the northern sector of the Campiglia area (Fig. 1), but its field relationships with respect to the other dykes are uncertain. It includes K-feldspar phenocrysts and, unlike the other felsic dykes, pristine biotite. Igneous activity in the area ended with the emplacement of the San Vincenzo Rhyolite, which consists of both fissural lava flows and lava domes emplaced above the Mesozoic Ligurian Units and the Pliocene sediments, and covering a surface area of ~ 10 km^2^ (Poli and Perugini^44^). The rhyolites are cordierite-bearing porphyritic rocks with glassy groundmass, with a variable content of mafic enclaves. Many authors concur that the San Vincenzo Rhyolite consists of two different, yet synchronous and coincident groups, which were identified on the basis of different mineralogical or geochemical features: Group-I and Group-II, the latter with lower Si, Li and ^87^Sr/^86^Sr ratios and higher LREE, Sr and Ba contents with respect to the former^45^; Group-A and Group-B, the latter characterized by the occurrence of ortho- and clinopyroxene as additional mineral phases, and lower ^87^Sr/^86^Sr and higher ^143^Nd/^144^Nd ratios with respect to the former^46^; low-Sr and high-Sr groups^47^; NGM and MG, not mixed and mixed groups^44^. Although the contact between the two groups is hitherto unknown, there is agreement that they display a different geographical distribution, with Group-A cropping out in the southern^46^ or in the southwestern sector^47^.

## Results

Minerals were separated for ^40^Ar–^39^Ar dating from selected samples of the whole plutonic-hydrothermal-subvolcanic-volcanic sequence, including: (1) biotite of the Botro ai Marmi Granite (sample BM05); (2) phlogopite of the exoskarn (sample PHLBM); (3) biotite and K-feldspar of the mafic Temperino porphyry (samples PV-31 and MGC); (4) K-feldspar or biotite from two types of felsic dykes, the Ortaccio (sample PV-51, K-feldspar) and the Monticino (PV-386, biotite) porphyries; (5) sanidine and/or biotite of different samples from the San Vincenzo Rhyolite: biotite from sample GZT4 (Group-A), and biotite and sanidine from sample SV85-6 and SV85-1 which were previously investigated by Ferrara et al.^46^ and belonging to Group-A and Group-B, respectively. Biotite separates from mafic porphyries were phenocrysts from the groundmass in sample MGC or grains included in a K-feldspar phenocryst in sample PV-31.

### (Micro)textural and microchemical data

**Figure 2:**
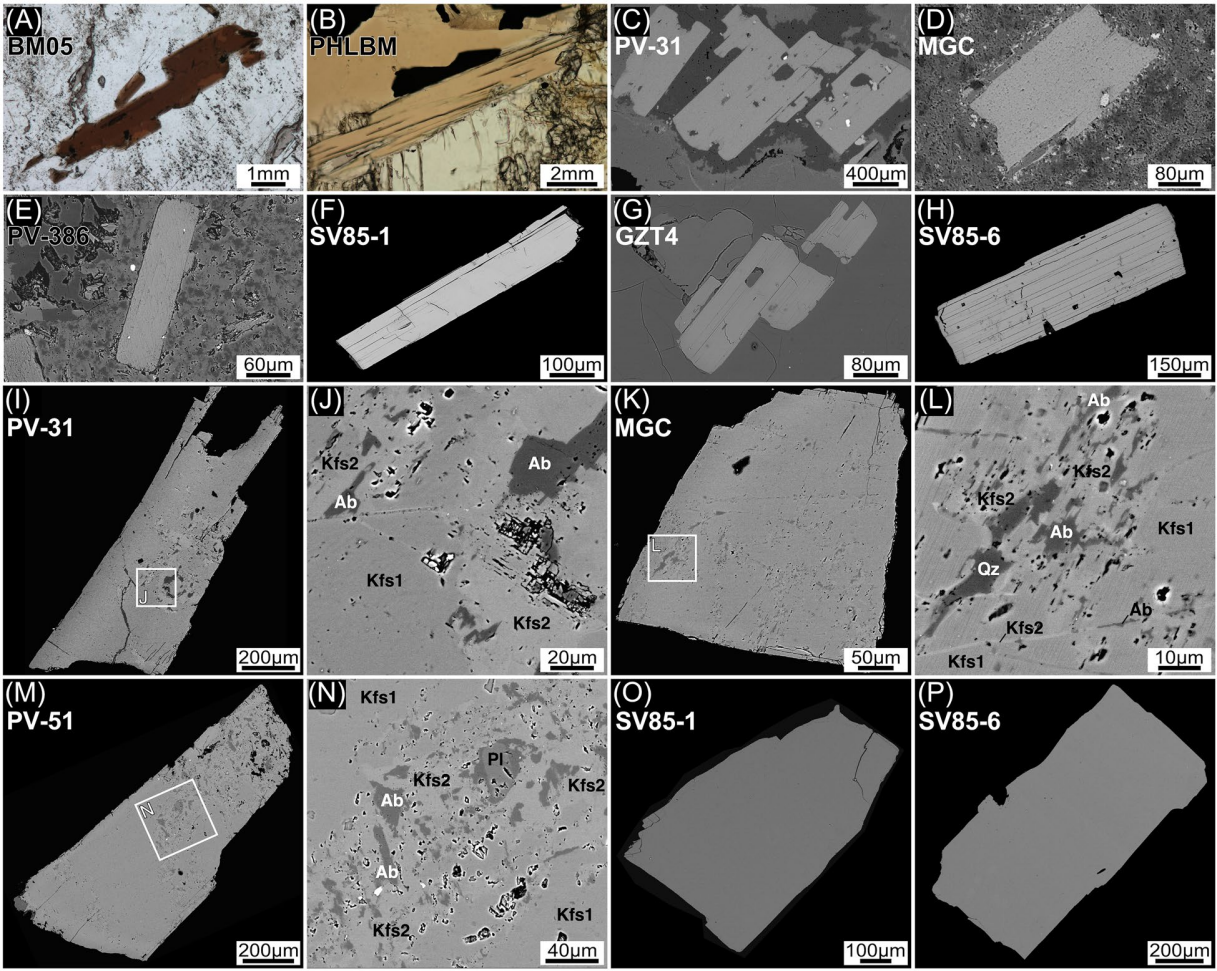
Photomicrographs (**A**,**B**) and backscattered electron images (**C**–**H**) of micas and K-feldpsar (**I**–**P**) from the different samples. (**A**) Botro ai Marmi Granite. (**B**) Exoskarn. (**C**,**D**) Mafic porphyries. (**E**) Monticino felsic porphyry. (**F**,**G**) San Vincenzo Rhyolite, Group-A. (**H**) San Vincenzo Rhyolite, Group-B. Backscattered electron images of K-feldspar grains in epoxy mounts. (**I**,**K**) K-feldspar from the Temperino mafic porphyry. (**J**,**L**) Close up of (**I**) and (**K**) respectively, showing alteration areas in igneous K-Feldspar (Kfs1) consisting of K-feldspar (Kfs2), albite (Ab), quartz (Qz). Note the occurrence of abundant microscopic pores closely associated to secondary minerals. (**M**) K-feldspar grain from the Ortaccio felsic porphyry showing similar internal microfeatures as K-feldspar from the mafic porphyries. (**N**) Close up of (**M**), plagioclase (Pl) likely corresponds to an original inclusion. Sanidine grains from the San Vincenzo Rhyolite, Group-A (**O**) and Group-B (**P**), characterized by the total absence of secondary alteration and inclusions.

**Figure 3:**
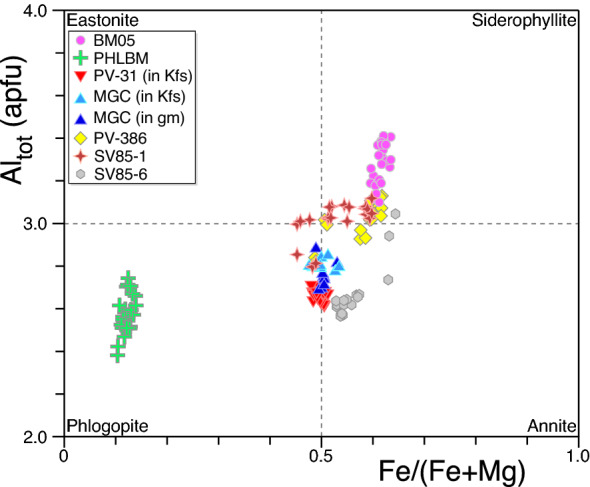
Compositional variations of trioctahedral micas [Al^IV^ vs Fe/(Fe + Mg) plot] from the Campiglia Marittima magmatic-hydrothermal system.

The Botro ai Marmi Granite is a medium-grained monzogranite, with K-feldspar phenocrysts (up to ~ 3 cm in length). The primary assemblage consists of quartz, K-feldspar, plagioclase, biotite and tourmaline, and accessory minerals cordierite, apatite, zircon and monazite. Unaltered monzogranite is only locally preserved due to a diffuse and pervasive hydrothermal K-alteration, testified by secondary K-feldspar replacing plagioclase and phlogopite replacing biotite^42^. Mafic enclaves have not been identified in the field. The selected sample (BM05) comes from a pristine decametric-sized granite lens in the altered main facies and includes pristine biotite. Biotite occurs as euhedral/subhedral (up to ~ 3 mm in size) pristine crystals (Fig. 2A). Rare, small and discontinuous chlorite interlayers were only occasionally observed. Electron microprobe (EMP) analyses (Supplementary Table S1) indicate a rather homogeneous composition (Fig. 3), with an average Fe/(Fe + Mg) atomic ratio of 0.61 ± 0.01 (± SD, n = 28).

The exoskarn mainly consists of diopside, garnet, phlogopite, amphibole, scapolite, vesuvianite, and wollastonite^48^. The selected sample is a phlogopite-rich exoskarn (PHLBM), composed by phlogopite and minor diopside and anorthite, and rare pargasite. The phlogopite has an isotropic texture, consisting of pristine crystals up to 1 cm in size (Fig. 2B). EMP data again reveal a rather homogeneous composition, with a constant X_Fe_ atomic ratio of 0.12 ± 0.01 (± SD, n = 29) and slight variation in Al^IV^ content (Fig. 3).

The mafic Temperino porphyry is a dark coloured porphyritic rock with phenocrysts of plagioclase, clinopyroxene, olivine, biotite, and abundant xenocrysts of quartz and centimetre K-feldspar with biotite, plagioclase, and quartz inclusions. The groundmass is completely transformed into a fine-grained aggregate of K-feldspar, quartz, and chlorite. Accessory minerals include chromite, apatite, zircon, monazite, and ilmenite. The mafic porphyries are variably altered, with mafic phenocrysts partially to totally transformed into amphibole (tremolite-actinolite group), epidote, Mg-rich chlorites and carbonates, and plagioclase partially replaced by secondary K-feldspar. Only biotite and rare clinopyroxene relicts have been observed as primary mafic phases. Selected samples (PV-31 and MGC, Fig. 1) were collected from the Gran Cava adit (Temperino mine). Sample PV-31 is dominated by a centimetre-sized K-feldspar (~ 6.5 cm long) containing biotite, and minor quartz, plagioclase, and zircon inclusions. Sample MGC, collected ~ 50 m away from the previous sample, contains biotite phenocrysts preserved in the groundmass. Biotite occurs as subhedral to anhedral crystals, up to ~ 2 mm in size. Back-scattered electron (BSE) imaging reveals the occurrence of pristine biotite with only rare and thin rims with alteration to chlorite (Fig. 2C,D). The biotite crystals, both those enclosed in K-feldspar xenocrysts (PV-31 and MGC) and those from the groundmass (MGC), are chemically homogeneous with a mean Fe/Fe + Mg atomic ratio of 0.50 ± 0.01 and an Al^IV^ of 2.49 ± 0.04 (± SD, n = 46) (Fig. 3). The two K-feldspar separates (samples PV-31 and MGC) show similar intragrain features, characterized by submillimetre turbid domains. BSE imaging and EMP analysis reveal that grains consist of igneous K-feldspar (Kfs1) with patchy alteration domains, from tens to hundreds micrometre in size, consisting of secondary K-feldspar (Kfs2) and albite (Ab) and minor quartz, typically accompanied by numerous micropores (Fig. 2I–L). The secondary assemblage replaces the igneous Kfs1 along randomly oriented veins, locally extending along cleavage/cryptoperthitic planes. The Kfs1 have lower K_2_O contents and higher Ca/K ratios than Kfs2. Secondary albite has a low K_2_O content (< 0.2 wt%) and Ca/K ratios > 1.5 (Supplementary Table S1).

Felsic dykes have porphyritic textures, with phenocrysts of quartz, sanidine, plagioclase, biotite, and cordierite, set in a fine-grained groundmass, totally transformed into K-feldspar, quartz, and minor chlorite. Pristine biotite is only preserved in the Monticino sub-unit. Samples from the southern area experienced intense hydrothermal alteration, testified by the total replacement of biotite by chlorite and by the partial replacement of plagioclase by K-feldspar. Studied samples are from the Ortaccio and Monticino sub-units, given that the Coquand dykes are pervasively altered. Sample PV-51 (Ortaccio porphyry) is characterized by abundant centimetre K-feldspar phenocrysts, with textural and chemical features similar to those of K-feldspar of the mafic dykes (Fig. 2M,N). Biotite from the studied sample of the Monticino porphyry (PV-386) is pristine and up to ~ 1 mm in size, with apatite and zircon inclusions (Fig. 2E). Similarly to biotite from the Botro ai Marmi Granite and from the mafic dykes, biotite PV-386 exhibits a small chemical variation, with X_Fe_ of 0.58 ± 0.03 and Al^IV^ of 2.57 ± 0.03 (± SD, n = 17; Fig. 3).

The San Vincenzo Rhyolite is a grey porphyritic rock with a glassy groundmass which includes plagioclase, sanidine, quartz, biotite and cordierite phenocrysts, and apatite, monazite and zircon as accessory minerals. Group-B rhyolites also contains clinopyroxene megacrysts, clinopyroxene–orthopyroxene or orthopyroxene–plagioclase clots, and mafic enclaves of latite composition^39^. GZT4 is a typical Group-B rhyolite sample (Fig. 1). Additional biotite and sanidine separates were obtained from two samples originally investigated by Ferrara et al.^46^: sample SV85-6 from Group-A and sample SV85-1 from Group-B rhyolites. Biotite is euhedral (up to ~ 3 mm in size) and homogeneous, lacking chlorite interlayering (Fig. 2F–H). EMP data reveal moderate variability (Fig. 3), with Fe/(Fe + Mg) ratio of 0.55 ± 0.06 (± SD, n = 29) in sample SV85-1 and 0.57 ± 0.04 (± SD, n = 35) in sample SV85-6. Sanidine grains in both samples are homogenous and free of inclusions (Fig. 2O,P).

### ^40^Ar–^39^Ar data

**Figure 4:**
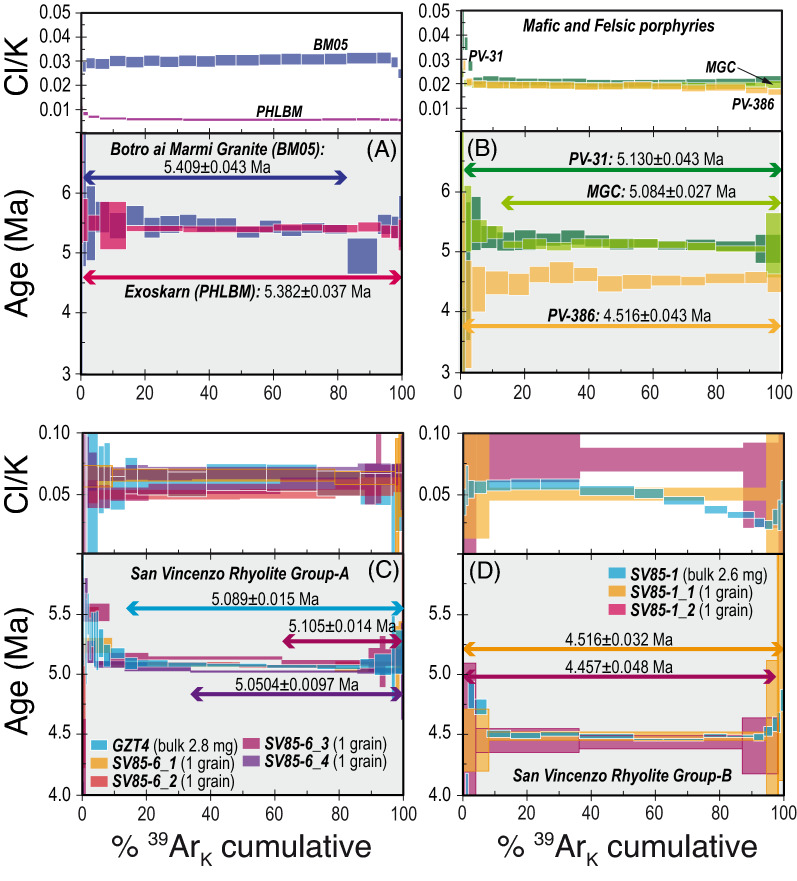
Age and Cl/K (derived from neutron-produced ^38^Ar_Cl_/^39^Ar_K_ ratio) spectra from step-heating experiments of trioctahedral mica separates. Box heights indicate the 2σ analytical uncertainty. (**A**) Biotite from Botro ai Marmi Granite and phlogopite from the exoskarn. (**B**) Biotite from mafic and felsic porphyries. (**C**) Multi-grain and single-grain step-heating experiments on biotite from Group-A of the San Vincenzo Rhyolite. (**D**) Multi-grain and single-grain step-heating experiments on biotite from Group-B of the San Vincenzo Rhyolite. Biotite analyses from Botro ai Marmi Granite, exoskarn and mafic and felsic porphyries were obtained on ~ 10 mg of mineral separate through a single-collector noble gas mass spectrometer. Data on biotite separates from San Vincenzo Rhyolite were obtained using a multi-collector noble gas mass spectrometer on single grains or on ≤ 3 mg of mineral separate.

Incremental heating data of trioctahedral micas from the Botro ai Marmi Granite, the exoskarn and the mafic and felsic porphyries, yielded concordant or nearly concordant age spectra (> 80% of the ^39^Ar_K_ released—Fig. 4A,B), characterized by constant Cl/K ratios (derived from neutron-produced ^38^Ar_Cl_ and ^39^Ar_K_) which, in agreement with BSE imaging and EMP data, attest to intra- and inter-grain homogeneity. Apparent ages from concordant segments strictly follow the relative sequence of emplacement as revealed by field data, ranging from 5.409 ± 0.043 Ma (± 2σ internal uncertainty) of the earliest igneous event, to 4.516 ± 0.043 Ma of the younger felsic Monticino porphyry. The age of Botro ai Marmi Granite is indistinguishable within internal uncertainties from the age of phlogopite from the exoskarn of 5.382 ± 0.037 Ma. Both ages are however significantly older then the two dates from the mafic porphyries, which gave concordant segments with indistinguishable ages of 5.130 ± 0.043 and 5.084 ± 0.027 Ma (Fig. 4B). Biotite from the San Vincenzo Rhyolite was analysed by the step-heating technique on milligram-sized aliquots (samples GZT4 and SV85-1) and on single grains (samples SV85-1 and SV85-6). Results revealed a more complex picture than expected. Samples from Group-A gave discordant age spectra, with overall descending shapes and variable total gas ages (~ 5.14 to ~ 5.05 Ma; Supplementary Table S2), and flat Cl/K spectra. Data from the multigrain aliquot and from two out of four single grains gave concordant segments (> 30% of the ^39^Ar_K_ released—Fig. 4C) from the intermediate- to high-temperature region, yielding heterogeneous mean ages from 5.105 ± 0.014 to 5.0504 ± 0.0097 Ma. The three runs completed on sample SV85-1 of Group-B yielded even more contrasting results. The two runs performed on single flakes gave similar results, with flat or nearly flat age spectra and indistinguishable mean ages at 2σ internal error of 4.516 ± 0.032 and 4.457 ± 0.048 Ma (Fig. 4D). The concordance is however only apparent, as the small size of analysed micas gave relatively large uncertainties which may obscure potential inhomogeneity. In fact, the multigrain aliquot yielded more precise results and a disturbed spectrum with an overall saddle shape (minimum step age ~ 4.48 Ma), with a total gas age of 4.5115 ± 0.0095 Ma. In agreement with these results, nineteen biotite grains, which were fused and analysed individually, yielded apparent ages ranging widely, from ~ 4.63 to ~ 4.47 Ma (Supplementary Table S2). It is important to note that apart from the intra-sample variability which requires scrutiny, biotite from Group-A gave apparent ages systematically and significantly older than those from group-B.

**Figure 5:**
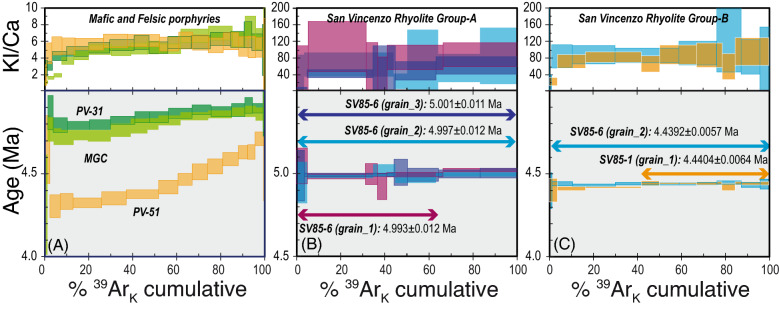
Age and K/Ca (derived from neutron-produced ^39^Ar_K_/^38^Ar_Ca_ ratio) spectra from step-heating experiments of K-feldspar separates. Box heights indicate the 2σ analytical uncertainty. (**A**) Mafic and felsic porphyries. (**B**) Sanidine from the Group-A of the San Vincenzo Rhyolite. (**C**) Sanidine from the Group-B of the San Vincenzo Rhyolite. Data for mafic and felsic porphyries were completed on ~ 10–15 mg of mineral separate, using a single-collector noble gas mass spectrometer. Data for San Vincenzo Rhyolites were completed on individual grains, using a multi-collector noble gas mass spectrometer.

**Figure 6:**
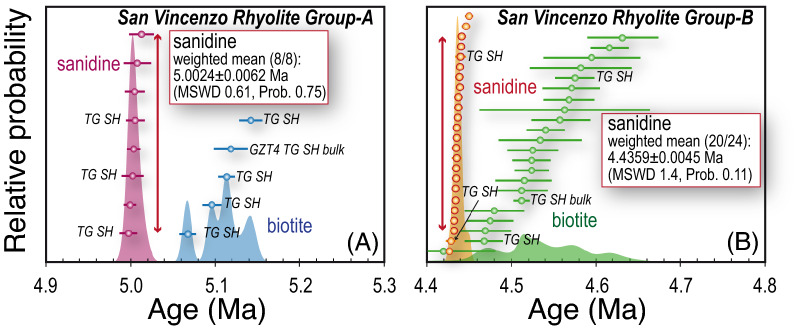
Cumulative probability and ranked distribution of ^40^Ar–^39^Ar apparent ages of sanidine and biotite from Group-A (**A**) and Group-B (**B**) of the San Vincenzo Rhyolite. Data are total fusion ages obtained from total fusion analyses of individual grains or calculated as total gas ages from step-heating experiments on single grains of both sanidine and biotite (TG SH) or on multigrain aliquots of biotite (TG SH bulk).

K-feldspar separates from mafic and felsic porphyries gave discordant staircase-shaped age spectra, accompanied by increasing K/Ca ratios (from neutron-derived ^39^Ar_K_/^37^Ar_Ca_) with increasing temperature (mafic dykes) or a nearly flat K/Ca spectrum (felsic dyke) (Fig. 5A). The two K-feldspars from the mafic porphyries gave similar apparent ages and K/Ca ratios, with ages ranging from ~ 4.73 to ~ 4.88 Ma and a similar maximum variation of 127 ± 60 ka and 78 ± 58 ka for MGC and PV-31, respectively. The felsic dyke (PV-51) gave significantly younger apparent ages (from ~ 4.31 to ~ 4.70 Ma), and a wider maximum age variation of 390 ± 50 ka (Fig. 5A). Sanidine of San Vincenzo Rhyolite were analysed by step-heating and total fusion analyses on individual grains. Three step-heating runs on sanidine from Group-A and two from Group-B gave concordant or nearly concordant age spectra, with indistinguishable within-group mean ages, in close agreement with the total gas ages (Fig. 5B,C and Supplementary Table S2). Five total fusion analyses on sanidine from Group-A gave concordant dates that combined with total-gas ages from step-heating runs yield a mean age of 5.0024 ± 0.0062 Ma (Fig. 6A). Nineteen out of twenty-two total fusion analyses on sanidine from Group-A gave indistinguishable ages with analytical uncertainties, with a pooled weighted mean age of 4.4359 ± 0.0045 Ma (Fig. 6B). Again, Group-A gave apparent ages significantly older than those from Group-B, and the intra-sample consistency of sanidine data strongly suggest that the two groups were emplaced at different times.

## Discussion

**Figure 7:**
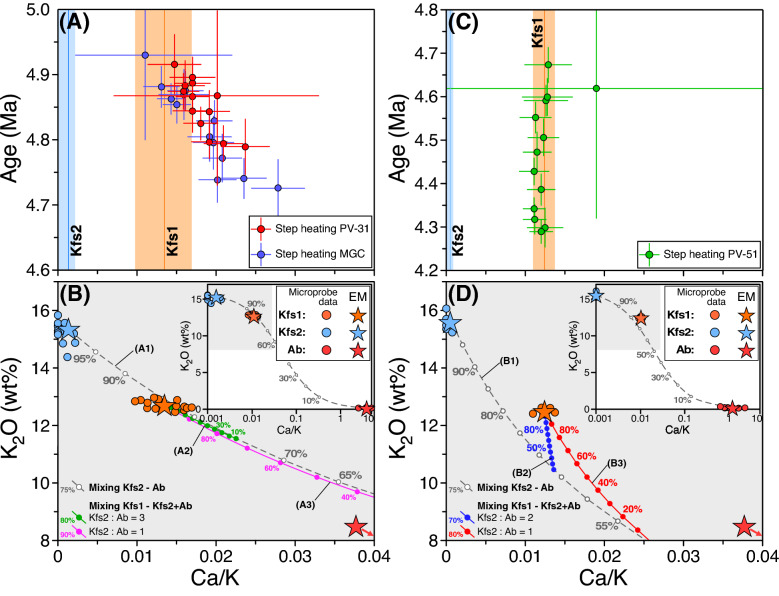
Three-isotope correlation diagram converted to age and elemental Ca/K ratio (derived from neutron-produced ^37^Ar_Ca_ and ^39^Ar_K_) for data from step-heating of mafic (**A**) and felsic (**B**) porphyry. K_2_O (wt %) vs. Ca/K (EMP data) of K-feldspar from mafic (**B**) and felsic (**D**) porphyries. The model lines in (**B**) and (**D**) show the effect of mixing secondary K-feldspar (Kfs2) and albite (Ab) with pristine K-feldspar (Kfs1). Model lines in (**B**): (A1) mixing between secondary K-feldspar and albite; (A2), mixing between pristine K-feldspar (Kfs1) with a mixture of secondary K-feldspar and albite in the proportion 3:1; (A3), mixing between pristine K-feldspar (Kfs1) with a mixture of secondary K-feldspar and albite in the proportion 1:1. Model line (**D**): (A1) mixing between secondary K-feldspar and albite; (B2), mixing between pristine K-feldspar (Kfs1) with a mixture of secondary K-feldspar and albite in the proportion 2:1; (B3): mixing between pristine K-feldspar (Kfs1) with a mixture of secondary K-feldspar and albite in the proportion 1:1. Stars in (B) and (D) indicate mean end-members compositions (EMP data) used in the mixing calculations.

The age sequence defined by ^40^Ar–^39^Ar dates from homogeneous and pristine minerals is in perfect agreement with the emplacement sequence revealed by field data, and age data for the onset of the Campiglia Marittima igneous-hydrothermal system remarkably matches available CA-ID-TIMS zircon U–Pb data for the Botro ai Marmi Granite^41,42^. We infer that retentive properties of trioctahedral micas were sufficiently high to allow minerals which escaped successive re-equilibration to record crystallization ages corresponding to the emplacement of the respective host rocks. Exceptions include ^40^Ar–^39^Ar data from K-feldspar of the quickly cooled mafic and felsic porphyries and results from biotite of the San Vincenzo Rhyolite. As for the former, sensitivity of K-feldspar to deuteric alteration is a well-known feature^49^ and these modifications have necessarily important consequences for the Ar isotope record. The staircase-shaped age spectra of K-feldspar affected by deuteric alteration has in fact been assigned to mixtures between heterochemical and diachronous K-feldspar generations^26^. K-feldspar from both mafic and felsic porphyries from the present study were affected by hydrothermal alteration, and gave overall younger ages with respect to coexisting biotite and discordant, staircase-shaped, age spectra, characterized by progressively older step ages along with increasing temperature, associated with increasing (mafic porphyries) or flat (felsic porphyry) K/Ca ratios. EMP data revealed for pristine K-feldspar lower K/Ca ratios when compared to secondary K-feldspar, that would be expected to produce a K/Ca spectra with an opposite trend, with decreasing K/Ca ratios along with increasing apparent ages. This feature requires involvement of an additional young phase with low K/Ca ratios, identifiable in secondary albite. The age vs K–Ca relationship of ^40^Ar–^39^Ar data from K-feldspar of mafic porphyries can be therefore explained by considering a three-component mixture, rather than a simply binary mixing, involving (1) pristine K-feldspar and hydrothermal (2) K-feldspar and (3) Na-feldspar. This is illustrated by the model lines of Fig. 7B, showing the result of mixing pristine K-feldspar (Kfs1) with different proportion of secondary K-feldspar (Kfs2) and albite (Ab). The trend observed in K-feldspar of mafic porphyries can be accounted for by mixing pristine K-feldspar with a mixture consisting of secondary K-feldspar and albite in the proportion of ~ 3:1 (curve A2 in Fig. 7B). Analogously, using appropriate proportions, it is possible to explain the nearly flat Ca/K ratio with increasing temperature in the step-heating experiment of K-feldspar of sample PV-51 (curve B2 in Fig. 7D). Even if the involvement of a three-component mixing precludes a straightforward constraint on the age of the two K-feldspar generations, step-heating data may be used to define the minimum time elapsed between the emplacement of the mafic-felsic porphyries and the end of hydrothermal K-feldspar alteration.

Results from San Vincenzo Rhyolite indicate that biotite yields older, heterogeneous and less precise ages than coexisting sanidine (Fig. 6). Older ages with respect to coexisting sanidine and discordant age spectra, may in principle arise from analytical artefacts due to recoil loss or redistribution of Ar isotopes during sample irradiation. Chlorite interlayers may produce discordant age spectra but back-scattered electron imaging and EMP data discount chloritization as an important factor. Firstly, chlorite interlayers were very rarely imaged at the submillimetre scale and, secondly, alteration of biotite to chlorite would have produced hump-shaped age spectra and anomalous younger total gas ages^50^. Recoil loss of ^39^Ar_K_ during sample irradiation may produce meaningless old ages^51^ but this effect does not appear significant in the present samples. The biotite grains used in this study were sufficiently thick (> 100 μm) to make recoil loss a negligible factor^52^, in agreement with the observation that biotite with similar thickness gave different apparent ages. The chronometric unreliability of volcanic biotite was previously documented by Radicati di Brozolo et al.^53^. Similarly to the results from the present work, Hora et al.^54^ found systematically older ^40^Ar–^39^Ar ages of biotite with respect to coexisting sanidine in samples from the Andean Central Volcanic Zone, with age discrepancy up to ~ 0.6 Ma. They assigned age discordance to a pre-eruptive extraneous Ar component (either excess or inherited Ar) hosted in biotite which did not affect coexisting sanidine. We infer that retentivity of biotite from the San Vincenzo Rhyolite was high enough to preclude its total re-equilibration with the parental magma at the time of eruption and, similarly to Hora et al.^54^, that biotite preserves a pre-eruptive extraneous Ar component, either (1) excess Ar, parentless ^40^Ar producing geologically meaningless old ages, or (2) inherited Ar, pre-eruptive radiogenic ^40^Ar recording the time of biotite formation. Results are currently insufficient to discern whether excess or inherited Ar was the main cause of discordance, progress on this matter requires further and dedicated studies in order to clarify the siting of extraneous Ar in biotite. Nevertheless, one observation is particularly noteworthy, namely age spectra were discordant, with descending shapes, even for step-heating runs completed on individual crystals, suggesting that the presence of excess Ar appears to be a more likely possibility. This hypothesis would be in line with the higher solubility of Ar in biotite compared to that in sanidine^55^. Data from the present study reiterate that significant caution is required when using biotite alone in geochronological studies of volcanic rocks, as biotite may include an unequilibrated pre-eruptive extraneous Ar component, which produces older dates than the time of eruption. In the Campiglia Marittima system this drawback was limited to volcanic rocks, and did not affect coexisting sanidine, and plutonic and sub-volcanic rocks. The single-grain total fusion technique turns out to be an effective and fast approach to reveal grain-to-grain inhomogeneity and ascertain the presence of a pre-eruptive extraneous Ar component.

### Timescale and lifetime of the Campiglia Marittima magmatic-hydrothermal system

**Figure 8:**
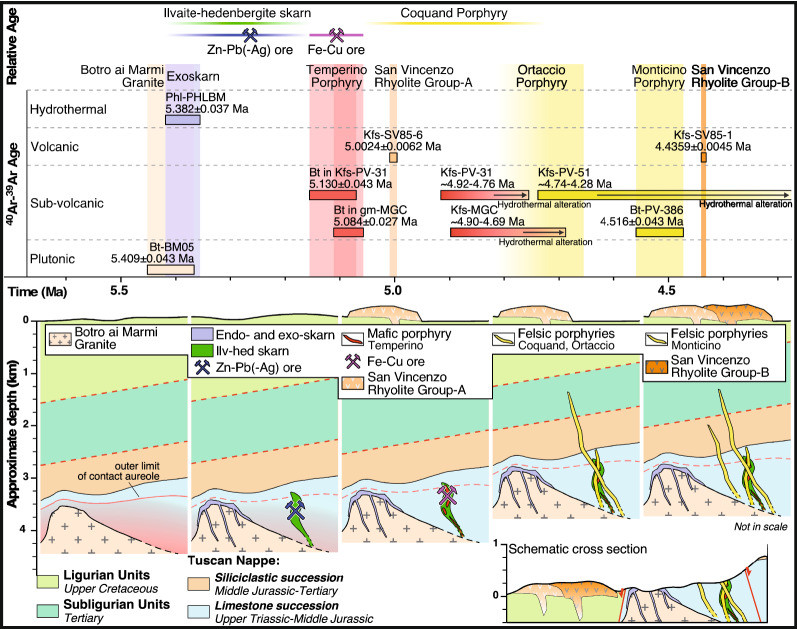
Sketch drawing summarizing the evolution of the Campiglia Marittima magmatic-hydrothermal system and consisting of intrusive, hydrothermal, subvolcanic and volcanic rocks.

^40^Ar–^39^Ar data from the different minerals, which were selected in order to cover the whole plutonic-hydrothermal-subvolcanic-volcanic sequence, constrain the duration of the Campiglia Marittima system to 973 ± 43 ka (Fig. 8). Activity began with the emplacement of the Botro ai Marmi Granite in the late Messinian (5.409 ± 0.043 Ma). The intrusion was closely followed by the formation of the exoskarn at 5.382 ± 0.037 Ma, that is after 27 ± 57 ka. The skarn was in turn followed by the emplacement of mafic porphyries, which began at 5.130 ± 0.043 Ma, after as much as 252 ± 57 ka. This indirectly constrains the emplacement of the ilvaite-hedenbergite skarn and the associated Zn–Pb sulphide ores to have occurred between ~ 5.38 and ~ 5.13 Ma. The two mafic dykes gave ages indistinguishable within internal uncertainty. The emplacement of the mafic porphyries induced overprinting of the Zn–Pb sulphides by Fe–Cu sulphide ores, this constrains emplacement of the last significant ore deposition to ~ 5.1 Ma. The emplacement of mafic dykes was followed by hydrothermal alteration which pervasively affected K-feldspar but produced undetectable effects on coexisting biotite. Based on the intermediate-temperature steps with lower K/Ca ratios from step-heating experiments on K-feldspar of mafic porphyries, fluid circulation able to modify the primary K-feldspar should have not ended before 399 ± 61 ka after the emplacement of the mafic porphyries (based on both ^40^Ar–^39^Ar data of coexisting biotite and K-feldspar). For the first time, results document that the San Vincenzo Rhyolite consists of two diachronally distinct batches, corresponding to Group-A and Group-B of the literature. Group-A rhyolites, the pure anatectic end-member^44^ emplaced at 5.0024 ± 0.0062 Ma and were followed at 4.4359 ± 0.0045 Ma, that is after 567 ± 8 ka, by the emplacement of Group-B rhyolites, representing anatectic melts variably contaminated by mantle-derived magmas^44^﻿. This implies that volcanism commenced much earlier than hitherto believed and that volcanic activity does not simply represent the final expression of the magmatic-hydrothermal system but it seems to accompany the whole sequence, with at least two main eruptions. The emplacement of Group-A rhyolites strictly followed, after 81 ± 28 ka, that of the mafic porphyries but likely preceded the emplacement of the Ortaccio felsic dyke and decidedly that of the Monticino porphyry. Group-B rhyolites were certainly associated with mafic magmas as testified by their hybrid nature and by the ubiquitous presence of mafic enclaves^44^. A cause-effect relationship between injection of primitive magmas at the base of a magma chamber and volcanic eruption has been previously proposed by Buret et al.^8^. They also showed that porphyry copper deposits and volcanic eruption may occur in close succession and that both are linked to the same mafic magma recharge event. We note that the bulk ore deposition in the Campiglia Marittima magmatic-hydrothermal system ended with the Fe–Cu sulphide ore at ~ 5.1 Ma, which was associated with and triggered by the emplacement of mafic porphyries. This implies that the first eruption should already have run out the ore-forming capacity of the crustal magma chamber involved in the system. The second and final eruption should have been favoured by a new replenishment of mafic magma at the base of the crustal reservoir, as testified by the strongly hybrid character of Group-B rhyolites, and definitively run out the system. Deuteric alteration of K-feldspar of the later felsic porphyries, continued until ~ 4.3 Ma.

## Methods

### Imaging and microchemical data of minerals

Minerals in polished thin sections and epoxy mounts (K-feldspar separates from samples MGC, PV-31 and PV-51, and K-feldspar and biotite from samples SV85-1 and SV85-6) were investigated by optical microscopy and by a scanning electron microscope (SEM), and analysed by the electron microprobe (Supplementary Table S1). Back-scattered electron imaging was acquired by a SEM Philips XL 30 equipped with an X-ray energy-dispersive system EDAX PV 9900 (at the Dipartimento di Scienze della Terra, Università di Pisa) and by a field emission SEM FEI Quanta 450 ESEM FEG, equipped with an energy-dispersive X-ray fluorescence spectrometer Bruker QUANTAX XFlash detector 6/10 (at the Centro per l’Integrazione della Strumentazione Scientifica dell’Università di Pisa—CISUP, Università di Pisa). Microchemical data were obtained by an automated JEOL 8200 Super Probe at the Dipartimento di Scienze della Terra Ardito Desio (Università di Milano). Operating conditions were: accelerating voltage 15 kV; beam current 5 nA; beam size 3 μm. X-ray counts were converted into oxides wt% using a PAP correction program^56^. Mineral standards were: grossular, fayalite, rhodonite, olivine, omphacite, K-feldspar, celestine, sanbornite, hornblende and scapolite. Counting times were 30 s for peaks and 10 s for backgrounds.

### ^40^Ar–^39^Ar data

^40^Ar–^39^Ar analyses were performed on mineral separates (biotite, phlogopite and K-feldspar) at IGG–CNR (Pisa, Italy), using both the laser step-heating and the laser total fusion techniques. Mineral separations were completed using standard separation techniques, followed by careful handpicking under a stereomicroscope. The recovery of sanidine from sample SV85-6 was very poor. K-feldspar was leached for a few minutes in an ultrasonic bath using HF 7% at room temperature. Mineral separates, after final cleaning by alternating methanol and deionized water, were wrapped in aluminium foil and irradiated along with either the Fish Canyon Tuff sanidine (FCs) or the Alder Creek sanidine (ACs) in the core of the TRIGA reactor at the Università di Pavia (Italy) in three distinct batches: (1) for 30 h, irradiation PAV-75; (2) for 2 h, irradiation PAV-82; (3) for 3 h, irradiation PAV-87. Argon isotope compositions were determined using either a MAP215-50 (Mass Analyser Products) single-collector noble gas mass spectrometer (irradiation PAV-75) or an ARGUS VI (Thermo Fisher Scientific) multi-collector mass spectrometer (irradiation PAV-82 and PAV-87). The neutron fluence was monitored by analysing single grains of the FCs or ACs, which were melted using a continuous wave CO_2_ laser (New Wave Research MIR10–30 CO_2_ laser system). Laser step-heating experiments were performed on mica separates using the laser beam generated by either a diode-pumped Nd:YAG laser (DQY-UNO-S, Quanta System, ~ 20 W maximum power, 1064 nm) or a fiber laser (RedEnergy G4 50W EP-Z, SPI Lasers, 1059–1065 nm). Biotite from sample GZT4 (PAV-82) and K-feldspars were laser step heated using the same CO_2_ laser as above. The laser beams were defocused to 2-mm spot size and slowly rastered over the mineral separate. Steps were carried out at increasing laser power until complete melting. Total fusion analyses of single K-feldspar (samples SV85-1 and SV85-6) and biotite (sample SV85-1) grains were performed using the CO_2_ and the Fiber laser, respectively. Argon isotope compositions for irradiation PAV-75 were acquired by peak jumping through a single-collector noble gas mass spectrometer MAP215-50, fitted with a secondary electron multiplier. Gas purification (10 min, including ~ 2 min of lasering) was achieved by two SAES AP10 GP MK3 getters held at 400 °C, one SAES C-50 getter held at room temperature and, only for micas, a liquid nitrogen cold trap. Blanks were analysed every three to four analyses. A polynomial function was fit to blanks analysed during the day of acquisition, and unknown analyses were corrected based on the time of measurement. Line blank variation is given in the Supplementary Table S2. More details about mass spectrometer analysis can be found in Di Vincenzo and Skála^57^. Argon isotope compositions for irradiations PAV-82 and PAV-87 were acquired simultaneously through a multi-collector noble gas mass spectrometer ARGUS VI. Ar isotopes from 40 to 37 were acquired using Faraday detectors, equipped with 10^12^ Ω resistors for ^40^Ar and ^38^Ar and 10^13^ Ω resistors for ^39^Ar and ^37^Ar. Faraday detectors were cross calibrated for the slight offset using air shots. ^36^Ar was measured using a Compact Discrete Dynode (CDD) detector. Before the acquisition of data from irradiation PAV-87, the mass spectrometer was upgraded with an additional amplifier equipped with a 10^13^ Ω resistor on cup L1 (^38^Ar). Gas purification (4 min, including ~ 1 min of lasering) was achieved using three SAES NP10 getters (one water cooled, held at ~ 400 C and two at room temperature) and, only for biotite separates, a cryogenic condensation trap using an ethanol-dry ice mixture. Blanks were generally monitored every two runs and were subtracted from succeeding sample results. Line blanks are given in the Supplementary Table S2. More details about mass spectrometer calibration and analysis can be found in Di Vincenzo et al.^58^. The correction factors for interfering isotopes from K and Ca were determined on K-rich and Ca-rich glasses and are listed in Supplementary Table S2. Ages were calculated using decay constants recalculated by Min et al.^59^, an atmospheric ^40^Ar/^36^Ar ratio of 298.56 ± 0.31^60^, and an age of 28.201 ± 0.046 Ma for the FCs^16^ and of 1.1848 ± 0.0012 Ma for the ACs^17^. Data corrected for post-irradiation decay, mass discrimination effects and blanks (relative abundances) are listed in Supplementary Table S2. Uncertainties on the ages from single runs are 2σ analytical uncertainties, including in-run statistics and uncertainties in the discrimination factor, interference corrections and procedural blanks. Uncertainties on the total gas ages and on error-weighted means also include the uncertainty on the fluence monitor (2σ internal errors).

## Supplementary Information

Supplementary Tables.

## Data Availability

All data generated or analysed during this study are included in this published article (as Supplementary Information files, Supplementary Tables S1 and S2).
